# Temperature and Relative Humidity Estimation and Prediction in the Tobacco Drying Process Using Artificial Neural Networks

**DOI:** 10.3390/s121014004

**Published:** 2012-10-17

**Authors:** Víctor Martínez-Martínez, Carlos Baladrón, Jaime Gomez-Gil, Gonzalo Ruiz-Ruiz, Luis M. Navas-Gracia, Javier M. Aguiar, Belén Carro

**Affiliations:** 1 Department of Signal Theory, Communications and Telematics Engineering, University of Valladolid, 47011 Valladolid, Spain; E-Mails: cbalzor@ribera.tel.uva.es (C.B.); jgomez@tel.uva.es (J.G.-G.); javagu@tel.uva.es (J.M.A.); belcar@tel.uva.es (B.C.); 2 Department of Agricultural and Forestry Engineering, University of Valladolid, 34004 Palencia, Spain; E-Mails: gruiz@iaf.uva.es (G.R.-R.); lmnavas@iaf.uva.es (L.M.N.-G.)

**Keywords:** estimation, prediction, Artificial Neural Networks (ANN), tobacco drying process, signal processing

## Abstract

This paper presents a system based on an Artificial Neural Network (ANN) for estimating and predicting environmental variables related to tobacco drying processes. This system has been validated with temperature and relative humidity data obtained from a real tobacco dryer with a Wireless Sensor Network (WSN). A fitting ANN was used to estimate temperature and relative humidity in different locations inside the tobacco dryer and to predict them with different time horizons. An error under 2% can be achieved when estimating temperature as a function of temperature and relative humidity in other locations. Moreover, an error around 1.5 times lower than that obtained with an interpolation method can be achieved when predicting the temperature inside the tobacco mass as a function of its present and past values with time horizons over 150 minutes. These results show that the tobacco drying process can be improved taking into account the predicted future value of the monitored variables and the estimated actual value of other variables using a fitting ANN as proposed.

## 1. Introduction

Tobacco farming is one of the main economic activities in agricultural areas of countries such as Brazil, China, and India [1]. During the last decades, a large number of technological innovations in this specific crop have been developed in diverse fields such as machinery, cultivation techniques, and drying techniques. Some advances in tobacco cultivation are the improvement of the health and quality of the tobacco plants by means of genetic modifications [2], the utilization of more effective fertilizers as a consequence of studies of their influence in tobacco growing and quality [3], or the employment of new machinery and techniques to irrigate and collect tobacco plants [4]. Furthermore, the tobacco drying process has been studied and improved by employing new drying strategies and new machinery in order to increase the quality of the dry tobacco and the overall energy efficiency of the process [5].

The tobacco drying process requires exhaustive monitoring of its representative variables to optimize it [6]. Monitoring systems are usually employed to acquire and store the most useful variables of a process. In the literature, these systems have been proposed to monitor atmospheric phenomena such as air pollution [7], livestock farms such as aviary production systems [8], or environments including rivers [9], oceans [10], and ecological systems [11]. In the field of agriculture, monitoring systems have been proposed for indoor locations such as greenhouses [12–14], and for outdoor agricultural processes including wine harvesting [15], olive growing [16], or any generic process [17].

Data stored by monitoring systems have been used to analyse the connection among the system's variables and to obtain a model of the monitored process [18]. These models can be defined by means of fuzzy-logic, probabilistic estimators, or Artificial Neural Networks (ANN) among others. Using fuzzy-logic, Schulz *et al.* [19] modelled water flow, Barros *et al.* [20] analysed different demographic and environmental models, Malins *et al.* [21] modelled the spatial extent of salinity on a farming land, and Papantoniou *et al.* [22] proposed a model of wind power for farm applications. Using probabilistic estimators, Cornford *et al.* [23] and Ng *et al.* [24] proposed different models to forecast precipitations, Holland *et al.* [25] modelled animal populations, and Trombe *et al.* [26] modelled offshore wind power fluctuations. Using ANN, Recknagel *et al.* [27] modelled algal growing, Smith *et al.* [28] predicted the air temperature, and Singh *et al.* [29] modelled water quality.

The aforementioned processing data tools have also been employed to control and predict variables in agricultural drying processes. Using fuzzy-logic, Yliniemi *et al.* [30–32] designed the control logic for a rotary dryer and Li *et al.* [33–35] developed and tested a control algorithm for microwave drying of vegetables. Employing probabilistic estimators, Banga *et al.* [36] designed a stochastic control algorithm using temperature and relative humidity as input variables. Using ANN, Yliniemi *et al.* [30,31] designed a system to identify the dynamics of the drying process in a rotary dryer, and Movagharnejad *et al.* [37] proposed a tomato drying model which fitted the experimental data more accurately than other mathematical models.

A system to estimate and predict the temperature and relative humidity in the tobacco drying process is proposed and tested in this work. This system acquires temperature and relative humidity data in different points of a tobacco dryer, and processes this data with an ANN in order to estimate and predict their value in other locations or in the future. The methodology of the article was composed by four stages: (i) a wireless sensor network (WSN)-based monitoring system was deployed in the drying installation to capture input variables, (ii) an artificial neural network was developed to estimate output variables using input variables, (iii) different experiments were performed to analyse the proposed methods, and (iv) experimental results were analysed to evaluate the proposed method.

## 2. Tobacco Drying

### 2.1. Materials

**Figure 1.**
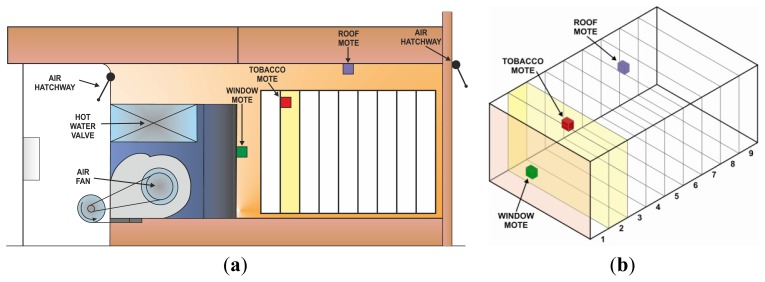
(**a**) Cross section of the tobacco dryer, where an air fan, a water valve, and two air hatchways are represented in their real locations. (**b**) Spatial distribution of the measurement motes inside the drying chamber: next to the supervision window (green), in the second container in the tobacco mass (red), and next to the dryer roof (purple).

A tobacco dryer from the Agrotex Company located in Rosalejo (Cáceres, Spain), was employed in this research. This tobacco dryer has a hot water valve to heat the air, a fan to move the air, and two air hatchways to remove the humidity. In this dryer, three measurement motes were deployed in its drying chamber to acquire the temperature and relative humidity in different points of the dryer. Figure 1(a) shows the schema of the cross section of this tobacco dryer, where the aforementioned equipment is represented. Figure 1(b) shows the spatial distribution of the three motes deployed inside the drying chamber: the first one was placed next to the supervision window (green), the second one was placed next to the roof (purple), and the third one was placed into the tobacco mass (red). The sensors of these measurement motes have been numbered as Sensors 1, 2, and 3, respectively, for easy reference.

A WSN with MEP510 motes from the Crossbow Company was employed to take the measurements. This mote has a SHT11-Digital Humidity Sensor from the Sensirion Company to acquire both temperature and relative humidity. LabVIEW and Matlab language programming tools were used to develop applications to acquire data from the WSN, to store it in a structured database, and to develop an ANN to estimate and predict these data.

### 2.2. Description of the Tobacco Drying Process

**Figure 2.**
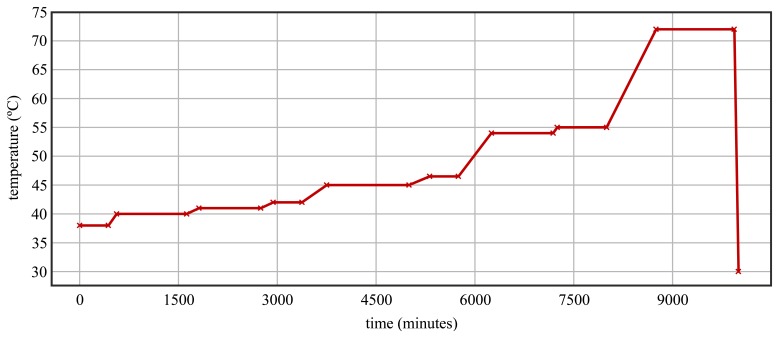
Target temperature evolution in the analysed drying processes.

The specific tobacco drying process monitored in this research was based on increasing the tobacco dryer chamber temperature in order to extract the plants' moisture. To this end, a time-varying target temperature as that shown in Figure 2, which is a typical target temperature evolution in this type of dryer [5], was chosen.

The drying process was directed by a general purpose microcontroller, which used a temperature sensor to acquire the instantaneous temperature next to the window measurement mote [Figure 1(b)] and set the output values of the heater according to an ON/OFF algorithm: if the dryer temperature is greater than the target temperature, the heater is turned off, and, if the dryer temperature is lower than the target temperature, the heater is turned on. Moreover, a hysteresis of 2 °C was employed in the control algorithm in order to avoid activating and deactivating the heater repeatedly. The target temperature evolution consisted of a concatenation of two types of phases: phases with a constant temperature, where the target temperature is invariable, and phases with a constant slope increasing temperature, where the target temperature increases progressively. These two types of phases are represented in the target temperature evolution shown in Figure 2.

### 2.3. Data Set

Nearly 900 hours (to be precise 53833 minutes) of drying process data were acquired. The temperature in each drying process varied between 20 °C and 75 °C, and the relative humidity range fluctuated between 10% and 100%. These data were acquired in the 2011 tobacco drying campaign, in the tobacco dryer presented in the Tobacco Drying section. An acquisition sample time of 3 minutes was used for every variable in all the drying processes, resulting in a total of 37 days containing 216,828 samples.

## 3. Artificial Neural Network for Data Completion

### 3.1. Aims and Purpose

The aim of this section was to design an ANN-based system for the estimation and prediction of temperature and relative humidity at different spatial points and with different time horizons inside a tobacco dryer, from the data sensed at a set of different meaningful points. This means that the system must carry out two different tasks:

- *Data Estimation*: from the readings of temperature and relative humidity obtained at a given moment *t* from a set of available sensors located at fixed positions inside the tobacco dryer (*input points*), the ANN must estimate the values of those magnitudes at a set of different spatial points (*target points*) at the same moment *t*. For that, the network was trained with the values of a single drying process for which the magnitudes at the target points are also known, but, once trained, it operates only with the sensors located at the input points.
- *Data Prediction*: from the readings of temperature and relative humidity obtained from a set of available sensors located at fixed positions inside the tobacco dryer at a set of *n* instants (*t-1*, *t-2, t-3*, …, *t-n)*, the ANN must predict the value of those magnitudes at the same spatial points at the moment *t* + *t_0_*. For that, the network was trained with the values of a single drying process. Once trained, it operates predicting the temperature and/or relative humidity of a process in *t*+*t_0_* using as input the temperature and relative humidity data at (*t-1*, …, *t-n)* interval.

### 3.2. Artificial Neural Network Design

Normally, when the data to be handled within an ANN is dependent on time, a straightforward solution is to use a time-series ANN [38,39], that is, a neural network with a delay architecture specifically designed to operate with an input which is a temporal sequence and capable of forecasting values of that sequence in the future.

However, in the case of this work, while the data to be handled is also a time series of temperature and relative humidity measures, another possible approach is to consider the drying procedure as a prototype pattern that will be replicated every other drying procedure. Using this approach, estimation and prediction are not made on the basis of the similarity and progression of values registered in moments near in time, but on the basis of the similarity of the input values with a canonical drying procedure pattern used for training. A fitting ANN is a good solution for implementing this approach. It is trained with the data available for one complete drying procedure and tested against the rest of the data.

Additionally, using a fitting network has the advantage of being more robust against failures in the sensors than a time-series ANN, especially for the *Data Estimation* task: if there is a failure in a sensor, in the case of the time-series network, all the predictions given by the ANN using that measure as an input fail. For instance, if the ANN uses the past *n* = *20* values of the input variables for its operation, a single missing or failing value will invalidate 20 predictions of the network. However, if a fitting ANN is employed for *Data Estimation*, one faulty input results in only one faulty output. This feature is highly desirable in environments like the one considered in this work where it is not unusual for sensors or sensor networks to fail.

Therefore, along this work, a fitting neural network was employed. The architecture of these networks was comprised by three neuron layers: an input layer, a hidden layer, and an output layer. The size of the input and output layers was determined by the number of inputs/outputs defined for each case, and this changes along this work according to the number of inputs/outputs employed in the different tests. The size of the hidden layer normally depends on the complexity of the data to be fitted. For this work, this size was set up at 20.

### 3.3. Trial Design

For each of the two tasks considered in this work, different tests were conducted in order to determine the performance of the ANN and find the influence of the different parameters in the accuracy of the output. For the *Data Estimation* task, the varying parameters considered were the following:

- Input magnitude: different tests were performed using only temperature, only humidity, or a combination of both as input data for the network.
- Number of inputs: different tests were performed using data from one sensor or combining data from two sensors as input.
- Output magnitude: two sets of tests were performed for *Data Estimation*, one of them estimating the temperature in one sensor, and the other estimating the humidity in one sensor.

For the *Data Prediction* task, the varying parameters considered are the following:

- Time horizon: different tests were performed to predict the values of a magnitude in different moments in the future: 15, 30, 150 and 300 minutes in advance.
- Length of input sequence: in order to perform the prediction, the length of the input sequence feed into the ANN was also varied.

**Figure 3.**
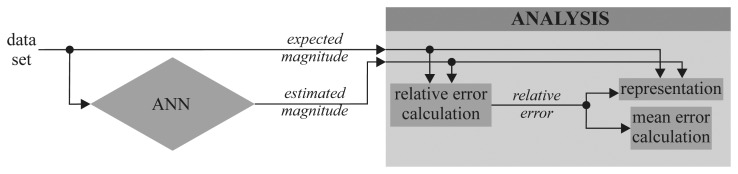
Diagram of the validation trials.

For numerical characterization of the results, a validation stage of the system was conducted in which its operation data were measured. Validation trials were carried out according to the diagram shown in Figure 3.

The relative error between the estimation and the measured magnitude value was obtained by means of Equation (1). Since the expected temperature was always greater than 0 °C (in fact it was always greater than 15 °C) there was no problem with the fraction denominator. The same formula was also applied to the calculations made for humidity:

(1) $$e[n](\%)=\frac{T_{\text{expected}}[n]-T_{\text{estimated}}[n]}{T_{\text{expected}}[n]}\cdot 100$$

### 3.4. Results

This section presents the numerical results of the test trials performed with the ANN. In all cases, the fitting ANN was trained with the data of a complete drying process and then tested against the rest of the data. With three sensors available and two magnitudes measured per sensor, data from Sensors 1 and 2 were always considered as potential inputs, and data from Sensor 3 was always considered as the target, that is, the data that the network predicts and estimates.

#### 3.4.1. Data Estimation

**Figure 4.**
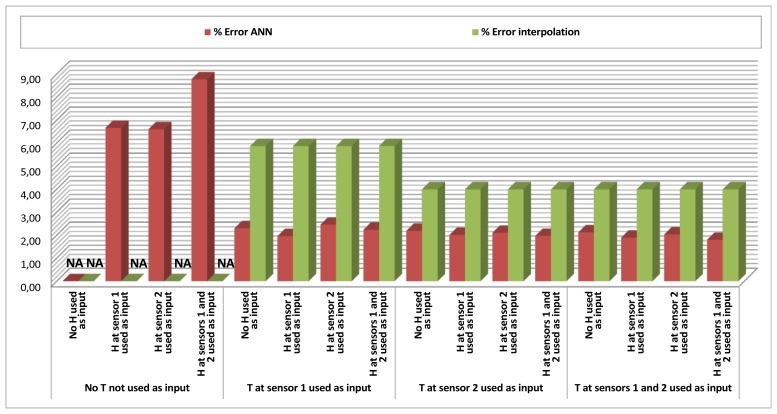
*Data Estimation* results (% error ANN/% error interpolation) for Temperature (T) in Sensor 3 (S3), combining Temperature (T) and Humidity (H) in Sensors 1 and 2 (S1 and S2) as inputs.

**Figure 5.**
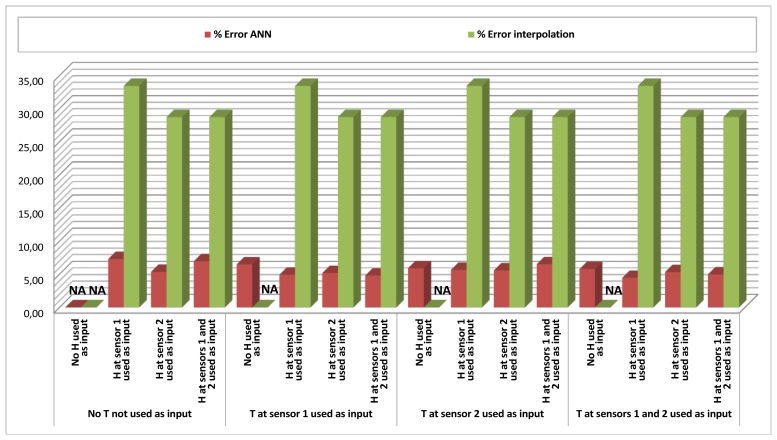
Data Estimation results (% error ANN/% error interpolation) for Humidity (H) in Sensor 3 (S3), combining Temperature (T) and Humidity (H) in Sensors 1 and 2 (S1 and S2) as input.

Tables 1 and 2, and their respective graphical representations in Figures 4 and 5, depict the results obtained for temperature and humidity *Data Estimation* at Sensor 3. This means that along this set of tests, the ANN was estimating the temperature (or humidity) given by Sensor 3 at moment *t* by using as an input a combination of data from temperature and humidity given by Sensors 1 and/or 2, also at moment *t*. Each cell of the tables represents a specific combination of inputs, and for each combination of inputs, 20 different tests were performed. The mean errors of the output against the real data were measured and averaged.

Additionally, for the cases when the input included a measure of the same magnitude to be estimated, a direct interpolation method was also implemented, in which the output (estimated value) was directly calculated as the input value of the same magnitude in a different sensor. This method assumed that points located in the vicinity of a sensor have similar measures for the same magnitude. The error of this interpolation method was also computed as a means to measure, by comparison, the improvement offered by the ANN estimation. In each of the cells of Tables 1 and 2, the leftmost value is the averaged mean error of the ANN's output, and the rightmost value is the averaged mean error of the simple interpolation method.

Looking at the results, it is easy to realize that when estimating temperature (Table 1, Figure 4), the ANN is roughly between two and three times more accurate (200%–300% improvement) than the simple interpolation method.

Another aspect to be noticed is that the results are not very accurate (mean errors around 6–9%) when only using humidity as an input (leftmost column of the table), but they demonstrate that a rough approximation to temperature can be made by the ANN when only humidity is available. However, even if humidity shows itself not to be sufficient to estimate temperature accurately, it contains information to improve the performance of the estimation when combined with temperature: the upper row shows results of cases when only temperature was considered as an input, and the lower rows, that combine temperature with humidity, generally show better results.

Looking at Table 2 and Figure 5 it is obvious that it is more difficult to estimate humidity than temperature. The best errors are around 5%, doubling those obtained for estimation of temperature. However, it is also obvious that an acceptable estimation of humidity (mean error of 5–6%) can be obtained only on the basis of temperature (upper row), and that the usage of the ANN in this case greatly improves the estimation based on interpolation, showing errors around six times smaller (errors around 5% against errors around 30%). Combined inputs of humidity and temperature render again the best results.

**Figure 6.**
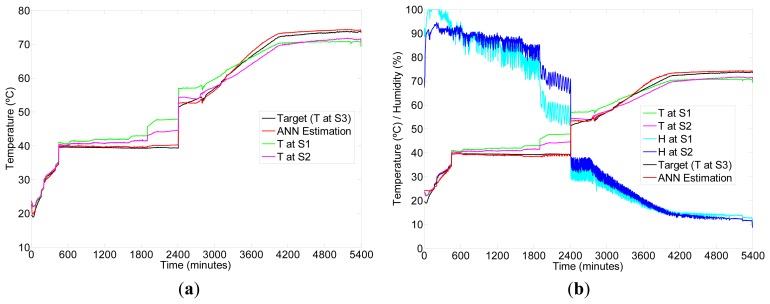
Example of ANN estimation of Temperature (T) at Sensor 3 (S3) for one drying process using Sensors 1 and 2 (S1 and S2) as input. (**a**) Input is only Temperature (T). (**b**) Input combines Temperature (T) and Humidity (H).

**Figure 7.**
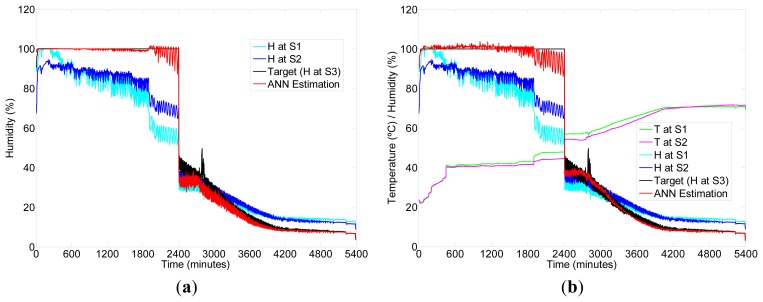
Example of ANN estimation of Humidity (H) at Sensor 3 (S3) for one drying process using Sensors 1 and 2 (S1 and S2) as input. (**a**) Input is only Humidity (H). (**b**) Input combines Temperature (T) and Humidity (H).

As an example, Figure 6 shows the results of a specific execution of the ANN estimation for temperature given by Sensor 3 during one drying session, using as input: (left) temperature at Sensors 1 and 2 and (right) temperature and relative humidity in Sensors 1 and 2. Figure 7 shows another specific execution of the ANN estimation for humidity, using as input (left) humidity at Sensors 1 and 2 and (right) temperature and humidity in Sensors 1 and 2. It is easy to see that for this specific session, there is a discontinuity in the data around 2,400 minutes as a consequence of a failure in the sensing hardware. However, the fitting ANN approach allows a successful estimation of values after the discontinuity, which is not the case when using a time-series based estimation.

#### 3.4.2. Data Prediction

**Figure 8.**
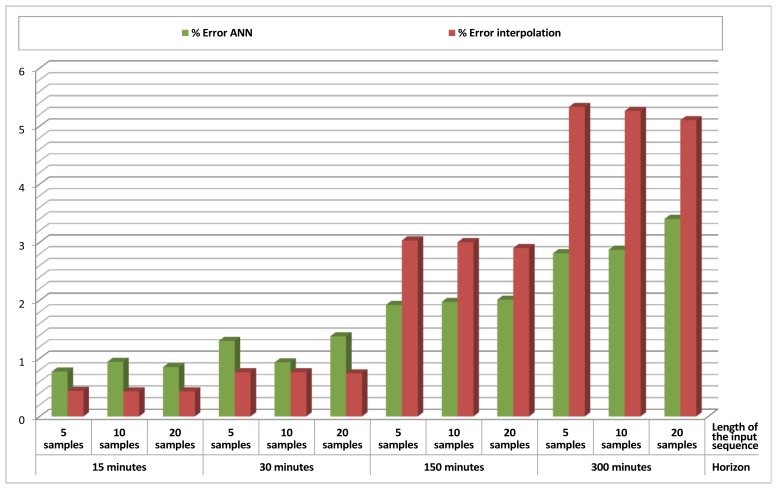
*Data prediction* results (% error ANN/% error interpolation) for temperature in Sensor 1, using temperature in Sensor 1 as an input.

Table 3 and its respective graphical representation in Figure 8 present the results obtained for *Data Prediction* of temperature in Sensor 1, using just its past values as an input. Different lengths for the input sequence (which contains values from *t-1* to *t-length_of_sequence*) and different time horizons (the value to be predicted is *t* + *time_horizon*) were considered and combined. For each cell of the table, 20 different tests were performed and the mean error of the output was measured and averaged. Again, an interpolation method was also implemented as a reference, using the value of temperature at *t* as the predicted value for temperature at *t* + *time_horizon*.

An analysis of these results shows that when the time horizon is short, even if the ANN gives small errors, the simple interpolation method performs better, giving extremely small errors. This happens because temperature varies relatively slowly, and thus recent values are normally valid for a very good prediction. However, as the time horizon increases, interpolation performs much worse, and errors obtained by the ANN are smaller than those obtained by interpolation.

These numbers also reveal that increasing the length of the input sequence could distort the prediction, demonstrating that saturating the ANN with data is not always a guarantee for better results.

**Figure 9.**
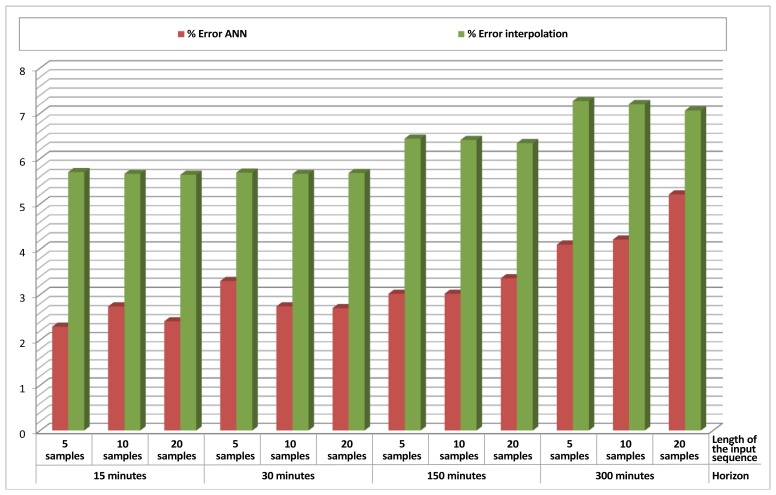
*Data prediction* results (% error ANN/% error interpolation) for temperature in Sensor 3, using temperature in Sensor 1 as an input.

Finally, Table 4 and Figure 9 present the results for the combined operation of *Data Estimation* and *Data Prediction*. In this scenario, temperature in Sensor 3 at a moment *t*+*time_horizon* was predicted on the basis of temperature in Sensor 1. This test was done to show that it is possible to use the ANN to perform both tasks at the same time, showing again good results when compared to interpolation.

## 4. Discussion

In light of the results, it is easy to realize that the use of the ANN results in a more efficient estimation and prediction task performance. For instance, for temperature estimation when there are no direct temperature readings in the system, the neural network offers a sufficient performance in the surroundings of 6–7% mean error, which is more than enough for an approximation; but even more, when there is temperature data, the ANN is capable of being between two and three times more precise than the interpolation method considered. When estimating the humidity, the results are even more meaningful, because even if the absolute mean error registered is around 5%, this error is between five and six times lower than the one obtained by interpolation.

The prediction task is slightly more complex, since, depending on the conditions, the performance of the ANN could greatly vary when compared to interpolation. Due to magnitudes varying slowly, it has to be considered that for small prediction horizons it might be better to directly interpolate the values. However, it has been shown that as the horizon increases, the neural network is capable of a more accurate prediction.

Estimations provided by the proposed system can be employed to detect sensors' failures and to recover their measurements when failures happen. Sensor failures can be detected comparing a threshold with the absolute difference between the estimated measurement obtained by the estimation method and the sensor measurement. If the absolute difference is higher than the threshold, then a failure has occurred. In this case, and until the sensor problem is solved, the measurements of this sensor can be replaced by the estimation provided by the ANN using the rest of the sensors measurements as input data. Moreover, this data recovering application can be used to recover data when sensors have other type of failures, such as sensor signal corruption or sensor signal loss, which are two typical problems in WSN [40,41].

Another application of the proposed estimation methods is the design optimization of data acquisition systems. To this end, a set of sensors must be deployed in several significant points of the monitored system. After that, the proposed estimation methods can be employed to discard the sensors whose measurements can be more accurately estimated using the rest of the deployed sensors with a low error. One advantage of these designed systems is their economy due to discarding the sensors whose measurements can be estimated with the other sensors of the system. Another advantage is that the measurements of sensors sited in a point with dangerous or hostile conditions that can quickly degrade the hardware can be replaced with the estimated measurements calculated from data of other sensors sited in safer places with better conditions.

Proposed estimation and prediction methods can be used to model processes. Models establish relationships among the variables of a process and predict the future evolution of these variables. They have been applied in the literature to plant growing processes [42–46], vegetal evapotranspiration processes [19,47,48], or drying processes [37,49,50] among others. One way to establish the relationships and to predict the evolution of processes' variables is using ANNs, which have been used to model algal growing [46] or drying processes as in the carrot drying process [51] and in the tomato drying process [37]. Process models are usually developed as a part of model-based predictive control (MPC) algorithms. MPC are multivariable control algorithms which use prediction methods to estimate the future state of the system and an optimization cost function in order to calculate the optimum control signals taking into account the predicted future state [52–63]. ANN-based prediction methods as that proposed in this article can be used in a MPC algorithm as the prediction method. ANN-based MPC algorithms have been proposed to control industrial applications [59–61], aircraft navigation [62], and laboratory processes [63].

Another application of the proposed data prediction methods is the improvement of alarm systems' performance. A typical problem in alarm systems is the high rate of false positives and false negatives. One cause of this problem is that alarm systems only take into account present data, or past and present data, so the incorporation of predicted future information about the system can improve the precision of the alarm system [64].

Finally, the proposed system's predictions can be easily employed to anticipate decisions about the drying process. The operator can see a forecast of the variables of the tobacco dryer in the future on the basis of the present conditions and also under a series of alternative conditions, making the process of taking the most appropriate decision to reach the desired values in the future much easier.

## 5. Conclusions

Tobacco drying has been traditionally a manually controlled activity, relying solely on experts' craftsmanship, and has been considered almost an art; however, as in many other areas of agriculture, new technologies have appeared as an easy solution to greatly improve the results and performance of the production processes while reducing costs at the same time. Specifically, introduction of sensor networks and intelligent automation systems represents a strong trend, as shown in the state of the art of drying processes. The performance of these systems depends heavily on the accuracy of the data provided by the sensor network, and as such, the better the data provided, the better the results.

The work presented in this paper has shown that by using a fitting ANN it is possible to accurately estimate the values of the different environmental magnitudes employed to control the drying process at different spatial points. The numerical results obtained present very low errors, which at less than 2% could even be below some sensors' accuracy.

Additionally, the system proposed is also capable of predicting the values of those environmental magnitudes with different time horizons. In this case, the results are dependent on the specific time horizon considered. For nearby time horizons, the mean errors given by interpolation are similar to those of the ANN system; however, for distant time horizons, the mean errors given by the ANN are much lower than those obtained by interpolation.

Finally, it is worth mentioning that both capabilities of the presented system are potentially very useful for improving the performance of automatic control of tobacco drying processes while at the same time reducing costs, by increasing the quality and quantity of available data.

## Figures and Tables

**Table 1.** *Data Estimation* results (% error ANN/% error interpolation) for Temperature (T) in Sensor 3 (S3), combining Temperature (T) and Humidity (H) in Sensors 1 and 2 (S1 and S2) as input.

**Input T**	**No T used**	**T at S1**	**T at S2**	**T at S1 & S2**
**Input H**				
**No H used**		2.30%/5.87%	2.19%/3.99%	2.12%/3.99%
**H at S1**	6.66%/NA	1.96%/5.87%	2.01%/3.99%	1.89%/3.99%
**H at S2**	6.60%/NA	2.45%/5.87%	2.10%/3.99%	2.03%/3.99%
**H at S1 & S2**	8.77%/NA	2.23%/5.87%	1.97%/3.99%	1.80%/3.99%

**Table 2.** Data Estimation results (% error ANN/% error interpolation) for Humidity (H) in Sensor 3 (S3), combining Temperature (T) and Humidity in Sensors 1 and 2 (S1 and S2) as input.

**Input T**	**No T used**	**T at S1**	**T at S2**	**T at S1 & S2**
**Input H**				
**No H used**		6.48%/NA	5.92%/NA	5.78%/NA
**H at S1**	7.30%/33.43%	4.95%/33.43%	5.66%/33.43%	4.46%/33.43%
**H at S2**	5.36%/28.72%	5.19%/28.72%	5.59%/28.72%	5.32%/28.72%
**H at S1 & S2**	6.97%/28.72%	4.80%/28.72%	6.51%/28.72%	4.98%/28.72%

**Table 3.** *Data prediction* results (% error ANN/% error interpolation) for temperature in Sensor 1, using temperature in Sensor 1 as an input.

**Horizon**	**15 minutes**	**30 minutes**	**150 minutes**	**300 minutes**
**Length of Input Sequence**				
**5 samples**	0.77%/0.44%	1.30%/0.76%	1.92%/3.03%	2.81%/5.33%
**10 samples**	0.94%/0.43%	0.93%/0.76%	1.97%/3.00%	2.87%/5.26%
**20 samples**	0.85%/0.43%	1.38%/0.74%	2.01%/2.90%	3.40%/5.10%

**Table 4.** *Data prediction* results (% error ANN/% error interpolation) for temperature in Sensor 3, using temperature in Sensor 1 as an input.

**Horizon**	**5 samples**	**10 samples**	**50 samples**	**100 samples**
**Length of input sequence**				
**5 samples**	2.29%/5.70%	3.30%/5.69%	3.02%/6.44%	4.10%/7.27%
**10 samples**	2.74%/5.66%	2.74%/5.66%	3.02%/6.41%	4.21%/7.20%
**20 samples**	2.41%/5.64%	2.70%/5.68%	3.36%/6.34%	5.21%/7.06%
